# Literature Review on Adsorption Efficiency of Paracetamol on Different Adsorbent Materials

**DOI:** 10.3390/ijms27020623

**Published:** 2026-01-08

**Authors:** Julia Makulec, Alicja Chrzanowska, Paweł Siudem, Katarzyna Paradowska

**Affiliations:** Department of Organic and Physical Chemistry, Faculty of Pharmacy, Medical University of Warsaw, Banacha 1, 02-097 Warsaw, Poland; s086074@student.wum.edu.pl (J.M.); s086009@student.wum.edu.pl (A.C.)

**Keywords:** adsorption, paracetamol, waste water

## Abstract

Paracetamol (acetaminophen) is one of the most widely used analgesics and antipyretics. Due to its widespread use, it is also one of the chief contaminants in surface water and wastewater, raising a significant environmental concern. Traditional wastewater treatment systems are ineffective at removing pharmaceutical residues, which makes it necessary to search for alternative methods. One of the promising techniques is adsorption which is valued for its simplicity, cost-effectiveness and high efficiency. This review provides an in-depth analysis of the adsorption efficiency of paracetamol on various adsorbent materials. The physical and chemical mechanisms of adsorption are discussed together with the factors affecting the efficiency, such as pH, temperature and ionic strength. Of the materials tested, activated carbon shows the greatest efficiency, but nanomaterials, biocomposites, clays and zeolites also give promising results. The potential of emerging materials, including modified silica, polymer-grafted nanocomposites and biosorbents derived from waste biomass is also explored. Special attention is paid to regeneration capabilities and environmental sustainability. The study emphasizes the importance of adsorption as a technique for enhancing the treatment of pharmaceutical wastewater and mitigating ecological risks.

## 1. Introduction

**Figure 1:**
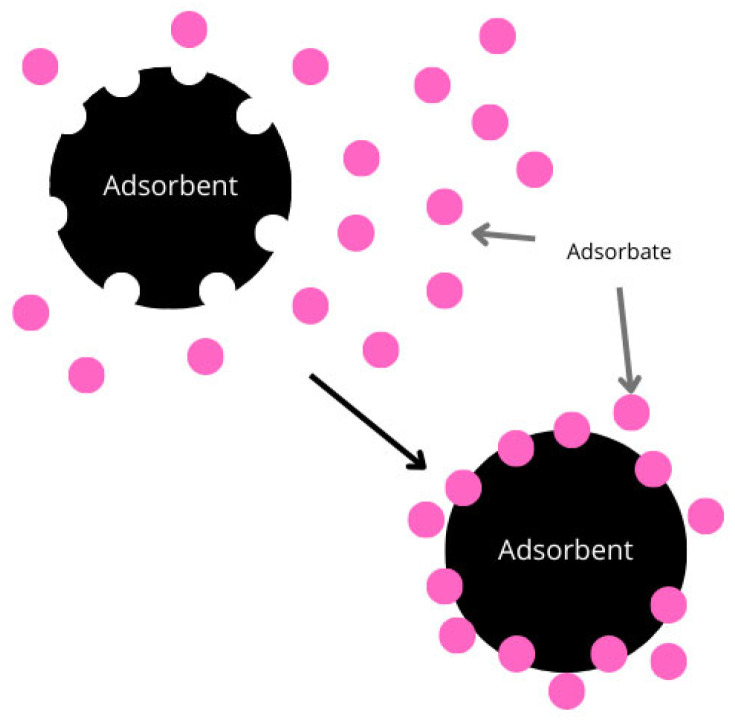
Adsorption scheme at the phase boundary.

The phenomenon of adsorption can be defined as the accumulation of substances dissolved in a liquid or present in the gaseous phase on the surface of a solid or a liquid. The adsorption process and the mechanisms that support it occur by adhesion of a gaseous, liquid, or solid material to the surface of a solid or a liquid (Figure 1). The solid or the liquid to which the material adheres is known as a sorbent or an adsorbent [1].

Adsorption can be categorized into different classifications, namely liquid–gas and liquid–liquid. In the case of a liquid adsorbent, the interfacial layer is designated a film, micelle, or emulsion. In the event of the adsorbent being a solid material, an alternative form of adsorption is observed, namely, solid–liquid or solid–gas. In such cases, the mechanism of the adsorption process is explained by the interfacial layer model. The resulting interfacial layer is indicative of the equilibrium between the adsorbent and the bulk phase. The first region is the substrate binding to the sorbent surface, and the second region is the surface layer of the sorbent. The interfacial layer mechanism can be explained by two principles. In the event of weak bonds between the substrate and the adsorbent, Van der Waals forces are the determining factor. No change in the chemical structure of either the substrate or the sorbent is observed. This phenomenon is known as physical adsorption. Conversely, in instances where chemical bonds are formed between the substrate and the adsorbent, whether by regrouping the electron density between the adsorbent and the substrate or by the formation of ionic or covalent bonds, we observe a process known as chemical adsorption, also referred to as chemisorption. In both cases, however, the appropriate adsorption model for the system is determined by experimental physical criteria. The physical criteria for comparing the two models under consideration are the thermodynamic or adsorption isotherm studies, as well as the kinetics or equilibrium adsorption studies.

**Figure 2:**
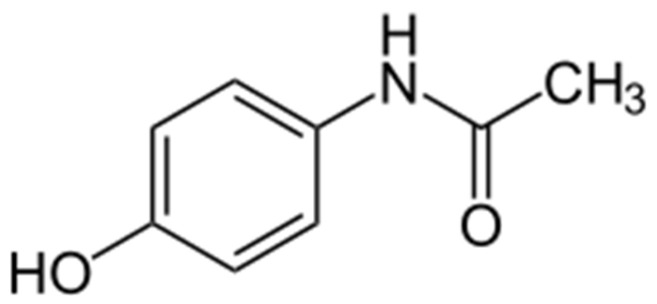
The chemical formula of paracetamol.

Paracetamol (also known as acetaminophen or 4-hydroxyacetanilide, Figure 2) is a widely used analgesic and antipyretic agent, available both by prescription and over the counter. The global demand for paracetamol is approximately 200,000 tons per year [2].

Paracetamol is available on the market under various brand names, in both simple and complex preparations. It was first synthesized in the 19th century [3] but it was not until the 1950s that it gained significant recognition when it was discovered to be a major metabolite of the highly nephrotoxic phenacetin [4].

From the chemical perspective, the substance is the hydroxyl derivative of acetanilide and its IUPAC nomenclature is N-(4-hydroxyphenyl)-acetamide.

The primary indication for paracetamol is to relieve mild-to-moderate pain of various etiologies and to lower a high temperature [5]. The above effects can be attributed to the mechanism of selective inhibition of type 3 cyclooxygenase found in the brain and spinal cord [6]. Furthermore, there are certain suggestions that the analgesic effect can be obtained due to attenuation of the pain stimulus by the activation of cannabinoid and vanilloid receptors [7]. Nevertheless, despite a nearly two-century presence on the market, the precise mechanism of action of acetaminophen is not yet fully elucidated. It is generally acknowledged that paracetamol exhibits no activity against COX-1 and COX-2, consequently lacking the capacity to impede the inflammatory process. Used in therapeutic doses, paracetamol is also practically devoid of gastrointestinal side effects that are characteristic of other non-steroidal anti-inflammatory drugs (NSAIDs) [8]. Because of that, it is considered by most patients to be a safe analgesic medication.

The organ that is predominantly (95%) responsible for its metabolism is the liver [9]. The primary mechanism by which paracetamol is inactivated involves its binding to glucuronates and sulphates. Furthermore, it was established that the substance undergoes conversion to N-acetyl-4-benzoquinimine by cytochrome P450, with the CYP2E1 isoform being particularly responsible for the process [8]. It was also found that metabolites of acetaminophen are excreted via the kidneys. The metabolic pathway and the mode of elimination from the body are associated with its nephrotoxicity and the risk of severe liver damage if toxic concentrations in the range of 3–30 mg% are reached or exceeded [10].

The development of pharmaceuticals has been a significant milestone in the advancement of science, contributing to the extension of life expectancy, the treatment of millions of individuals afflicted with serious diseases, and the enhancement of overall quality of life. The increasing importance of these substances as rapidly spreading environmental pollutants is directly related to their success [11]. This pollution constitutes one of the most significant environmental issues of global concern. The presence of pharmaceutical compounds in surface and groundwater is an indisputable fact. Consequently, there is an imperative for the effective removal of these pollutants from wastewater, as these molecules pose a grave threat to biodiversity and human health even at low concentrations. The article presents a range of removal techniques that may prove effective, depending on the target compounds and characteristics of the wastewater. However, a review of the extant research suggests that adsorption may offer a slight advantage due to the low cost of the adsorbent and operation, as well as its high efficiency and minimal by-products. The combination of physical, chemical and biological treatment methods has been demonstrated to be an effective approach for the removal of contaminants from wastewater.

## 2. Environmental Contamination Due to Paracetamol

Because of its pervasive use, paracetamol (acetaminophen) has emerged as one of the most prevalent pharmaceutical compounds in wastewater, with concentrations up to 6 μg in European wastewater treatment facilities [12]. Moreover, there is a deficiency of effective mechanisms for the elimination of paracetamol and its metabolites from the sewage system, resulting in the presence of those substances in surface water. For instance, concentrations of up to 10 μgL^−1^ were detected in surface water in the USA and levels exceeding 65 μgL^−1^ were recorded in the River Tyne in the UK [13]. It should also be noted that due to the abovementioned water contamination, the presence of paracetamol in aquatic organisms is a relatively frequent occurrence. The potential consequences of water contamination extend beyond the mere ecosystem complaint, as it can also lead to unpredictable and potentially hazardous metabolic reactions, which can result in the formation of structures with undefined pharmacological effects. Furthermore, the extensive presence of other drugs and their metabolites was found to increase the probability of drug–drug interactions [12]. Stuer-Lauridsen et al. [14] reported that the PEC/PNEC ratio (the ratio of the predicted concentration of a substance in the environment to its predicted concentration that would not cause adverse effects) for paracetamol exceeded 1, which indicates that the substance poses a risk to the ecosystem. The authors of this text are also concerned about the possibility of accumulation of other pharmacologically active substances in water-dwelling organisms. In 2006, Bound et al. obtained similar results [15]. They examined the concentrations of five common pharmaceuticals (paracetamol, ibuprofen, salbutamol, mefenamic acid, and propranolol) at the point of inflow of liquid effluent to sewage treatment plants in south-east England and in rivers downstream of those sites.

According to the classification system applied by the European Chemicals Agency (ECHA), paracetamol is categorized as a substance that is harmful to aquatic organisms, causing severe eye irritation, skin irritation, and potentially respiratory irritation [16].

It may seem that the issue of treating wastewater with pharmacologically active substances was already identified at the turn of the 20th and 21st centuries and it could be argued that the solution has now been found. Nonetheless, despite the implementation of a more efficient and environmentally oriented system for the treatment of industrial waste, the effectiveness of this system remains unsatisfactory. Therefore, in Directive No. 2024/3019 of 27 November 2024, in regard to the management of urban wastewater, the European Parliament and the Council of the European Union determined that wastewater arising from the production of pharmaceuticals and cosmetics necessitates a fourth-stage treatment [17,18]. Furthermore, it was stipulated that the manufacturers shall assume financial responsibility for the implementation and oversight of those measures.

The presence of a large number of contaminants in water and the detrimental effect they have on human health necessitated the development and improvement of water treatment methods [19,20,21]. In addition to the use of chemical methodologies (e.g., oxidation, coagulation, flocculation, or electrocatalysis) and biological methods (e.g., biodegradation or treatment with microorganisms), physical methods such as filtration, sedimentation, flotation, and adsorption were also applied [22]. Among them, adsorption is notable for its simplicity, cost-effectiveness and high efficiency (up to 99.9%), which means it is a highly effective method to deal with impurities.

Chemical treatment encompasses a range of processes, including advanced oxidation processes (AOPs), which, according to the extant data, are effective in the removal of antiviral/antibiotic drugs [23,24]. However, the implementation of these processes is costly and predominantly occurs in laboratory settings. The utilization of two alternative chemical methodologies, namely chemical oxidation and electrochemical processes, has the potential to yield by-products that are more noxious and toxic than the primary compounds present in wastewater. Another group of treatment methods is that of biological methods. Nevertheless, they are regarded as being ineffective and too time-consuming, a phenomenon that is related to the difficulties microorganisms have in breaking down antibiotics. Physical treatment methods encompass sedimentation, sand filtration, adsorption and membrane treatment [25].

The initial two processes (namely, sedimentation and sand filtration) are inherently incapable of processing pharmaceutical substances due to their design, which is optimized for the treatment of organic compounds with a concentration of mg/L. Membrane purification and nanofiltration, which have been shown to remove up to 85% of anti-inflammatory substances from wastewater, have been demonstrated to be highly effective in the removal of pharmaceutical compounds. However, it should be noted that the implementation of these methods can incur high financial costs. The financial outlay is increased by two factors. Firstly, there is the issue of frequent clogging of filters, and secondly, there is the need to clean them. Adsorption has been a subject of considerable discussion in recent years with regard to the reduction in micropollutant introduction into the environment. However, it should be noted that these alternative systems also have disadvantages. These include the time-consuming nature of the application process, the stringent operating specifications, the financial cost, and the need for periodic maintenance [26,27,28,29,30].

Notwithstanding the aforementioned disadvantages, adsorption technology is extensively utilized on account of its cost-effectiveness, ease of use, effectiveness and stability in the removal of pharmaceutical waste. In the preceding two-year period, approximately 500 articles have been published on the use of adsorption to remove pharmaceuticals from wastewater. At present, a plethora of adsorbents can be categorized across a multitude of scales, ranging from macro to nano. A substantial corpus of research has been dedicated to the removal of pharmaceuticals using various adsorbents, including activated carbon, biochar, porous carbon, zeolite, MOF, graphene, polymer, and perovskite [31,32]. The purification of pharmaceuticals using adsorbents is a straightforward process that requires minimal energy and is more readily applicable in real-life conditions. Adsorption-based purification of pharmaceutical wastewater is already in use.

Adsorption is defined as the process of binding of molecules (adsorbate) to the surface of an adsorbent (also termed a substrate) as a consequence of molecular interactions between them. In the event of the adsorbent possessing both high adsorption capacity and a high degree of affinity for the contaminant, it will be capable of binding the contaminant and thus removing even trace amounts of it. Adsorption isotherms, defined as the relationship between the amount of a substance adsorbed on the surface of an adsorbent and its concentration (or pressure) in a liquid at a constant temperature, are used to ascertain the effectiveness of the adsorbent. This facilitates the determination of the settling behaviour of the adsorbate on the adsorbent under varying concentration conditions. In the context of contaminants, the Langmuir-Freundlich isotherm is the most frequently applied model [33]. Adsorption was found to have certain advantages over other methods, such as coagulation and flocculation. For instance, it was shown that no by-products were generated, which is an important consideration in waste management. This is in line with the principles of Green Chemistry.

## 3. Adsorbent Materials Used for the Adsorption of Paracetamol

The functioning of adsorbent materials in the process of adsorption is characterized by adhesion of a liquid to the surface of the sorbent, resulting in the formation of a thin film. Adsorbent sorbents attract the liquid to their surface due to forces of molecular attraction. Multiple materials are used to adsorb paracetamol from the environment. Table 1 summarizes the advantages and disadvantages of the adsorbents described in the following chapters.

In addition to comparing the advantages and disadvantages of the indicated adsorbents, it is also worth summarizing the measurable data, i.e., adsorption capacity, the effect of pH on adsorption, and relative cost. This data is summarized in Table 2.

In the context of paracetamol, the pH of adsorption is a critical factor in determining the efficacy of the process. The phenomenon has the capacity to impact both the ionization of paracetamol and the surface of the adsorbent. Paracetamol is a weak acid (pKa ≈ 9.5) [50]. In conditions of acidic to neutral pH, paracetamol thus exists in a neutral form. During the process of adsorption, the formation of hydrogen bonds, π–π interactions (between aromatic rings and carbonaceous adsorbents), and hydrophobic interactions will be the predominant forces. A significant number of adsorbents (e.g., activated carbon, biochar, carbon nanotubes, biopolymers) demonstrate maximum uptake at a neutral pH level, which is the most favourable pH range for paracetamol adsorption in the majority of studies. In the context of a weak alkaline reaction, paracetamol may undergo partial ionization, thereby weakening hydrogen interactions. However, at a strongly alkaline pH, paracetamol occurs in an ionized form (negative charge), and if the point of zero charge is exceeded, the adsorbent also has a negative charge on its surface. Electrostatic repulsion is observed when the adsorbent surface is also negatively charged. Furthermore, in its ionized form, paracetamol exhibits enhanced solubility in water, thereby facilitating resorption [50].

### 3.1. Activated Carbon

Activated carbon is a mixture of asymmetrically distributed layers of elemental carbon and finely crystalline graphite. The presence of characteristic pores is attributed to two possible mechanisms: one is the coexistence of heteroatoms with carbon atoms, and the other is the formation of breaks in the crystal lattice. The process of adsorbent molecules filling the pores is contingent upon the diameter (d) of the aforementioned pores. Dubinin’s classification system distinguishes the following categories:

- micropores, defined as pores with a diameter of less than 2 nm, characterized by the volumetric penetration of molecules;
- mesopores, defined as having a diameter between 2 and 50 nm;
- macropores (d > 50, characterized by the phenomenon of capillary condensation.

The treatment of domestic and hospital wastewater using this method was shown to be an effective solution for the removal of a variety of pharmaceutical compounds, including paracetamol [51].

An additional advantage of activated carbon is its plant origin and environmentally friendly extraction methods (carbonization), as well as the possibility of regeneration and reuse [52]. The regeneration of pollutant absorption properties can be achieved by a number of methods. The most prevalent method involves the thermal burning of an organic adsorbent in special furnaces, with the organic adsorbent being derived from the pores of carbon [53]. However, this method has two significant drawbacks: firstly, there is a loss of mass of the sorbent, and secondly, once inorganic particles such as heavy metals are bound, complete cleaning of the pores is not possible [54]. Although a number of alternative regeneration methods were proposed, including washing with distilled water, extraction with ethanol, and carbon dioxide extraction with ethanol under supercritical conditions, they were shown to be significantly less efficient than the traditional firing methods [54]. Only vacuum annealing was found to exhibit significant efficiency. However, all of the aforementioned methods require significant resources, first of all, time and skilled personnel, so it seems reasonable to ask if the effort to regenerate the sorbent should be undertaken when fresh carbon can be produced from environmentally friendly sources.

Lignocellulosic materials, i.e., the precursor raw material for the manufacture of activated carbon, can be obtained from, for example, hemp [55]. The sorbent properties of this particular source were investigated by Macías-García et al. [56]. The material used was kenaf (K), from which activated carbon was prepared by chemical activation. The kenaf stock was impregnated with phosphoric acid (V) H_3_PO_4_ (at varying concentrations: 36%, 60%, and 85%). The reaction was carried out for two hours at a temperature of 85 °C. The final product was subjected to a thorough cleansing process involving distilled water (pH = 7) and subsequently dried [56]. The carbon thus obtained was then used for the purification of paracetamol-contaminated water, a process that unfolded in accordance with the kinetics of the pseudo-second-order model, encompassing two distinct stages. The initial stage was characterized by a rapid binding process that occurs on the surface of the adsorbent, whereas the subsequent stage focused on the binding within the pores. The analysis of the results was performed by the Fourier transform infrared spectroscopy (FTIR), a method used to identify the functional groups present within the molecule. The most favourable results were obtained with carbon activated with 60% H_3_PO_4_. Numerous studies showed that the presence of a significant number of oxygen-containing groups was conducive to the adsorption process [56]. Consequently, it is a suitable material for the fabrication of filters that treat wastewater contaminated with pharmaceuticals, particularly paracetamol.

Another study, described according to pseudo-second-order kinetics, was one in which three commercial adsorbents were analyzed [57]. Two of them were activated carbons (CAT and CARBOPAL), whereas the third one was silica gel. The adsorption process of paracetamol was carried out in two distinct environments, at a pH of 3.7 and 10.5, and at increasing levels of ionic strength (I) (0.01, 0.5 and 1M). Ionic strength can be defined as a measure of the inter-ion interactions present in solution. It should be noted that this measure determines the effect of all ions present in solution on their behaviour as well as their interaction with the electric field. The maximum binding capacities of the drug molecules were obtained for all three products when assays were performed at 25 °C, pH = 3 and I = 1 M. The values obtained for them were, respectively: the concentrations of CAT and CARBOPAL were 560 mg/L and 450 mg/L, with a silica concentration of 95 mg/L [57]. The following essay will provide a comprehensive overview of relevant literature on the subject.

Another research was performed on the use of activated carbon extracted from mango seeds. The subject was to ascertain the most favourable conditions for the purification of an aqueous solution contaminated with acetophenone [58]. The highest yields of the process, reaching up to 94.01%, were obtained using the following parameters:

- paracetamol concentration 150 ppm;
- amount of adsorbent used: 1.95 g;
- contact time of the adsorbent with the solution: 64 min.

The determination of the kinetic model of the Freundlich isotherms as the optimal fit was enabled by the correlation coefficient R^2^, which yielded a value of 0.972. That finding made it possible to corroborate the hypothesis concerning the multilayer development of molecular bonds within carbon pores. Furthermore, it was established that one gram of carbon obtained from mango seeds could bind 7.23 milligrams of paracetamol in an aqueous solution [58].

All of the above mentioned studies confirmed the ability of activated carbon to adsorb paracetamol and showed its significant advantage over other types of adsorbents. Therefore, their use in wastewater filters seems to be a reasonable measure, although the mechanism of adsorbent revitalization still needs to be improved to make the pharmaceutical waste treatment process more environmentally friendly.

However, activated carbon is a broad concept and can be obtained from various raw materials. The adsorption of paracetamol on carbon is known to vary in efficiency depending on the origin and method of preparation of the activated carbon [59]. Examples of differences in the use of different raw materials and activation methods are shown in Table 3.

### 3.2. Zeolites and Clays

Zeolites are hydrated aluminosilicate minerals with a cage-like structure. They possess both internal and external surface areas of up to several hundred square metres per gram, and a cation exchange capacity of up to several milliequivalents per kilogram [66]. The crystal structure of these compounds consists of tetrahedra of aluminum (Al) and silicon (Si) atoms with oxygen atoms linked via covalent bonds, forming a three-dimensional arrangement. These channels and pores facilitate the absorption and retention of water molecules and ions. The excess negative charge of zeolites is attributable to the substitution of some Si^4+^ cations by Al^3+^, a process that is compensated by cations such as Na^+^, K^+^, Ca^2+^, and Mg^2+^. These cations can be exchanged for other positive ions present in the environment. The process of water treatment is facilitated by ion exchange that occurs within the zeolite structure when water molecules enter or exit the structure. This mechanism involves the replacement of ions present in the surrounding solution with those found within the zeolite mineral. In addition to their use as an adsorbent material, both natural and synthetic zeolites are used as molecular sieves and ion exchangers.

Natural Jordanian zeolite was found to be efficacious in the removal of pharmaceuticals [67]. The examined material was characterized by a substantial surface area, microporous structure, and high adsorption capacity. Regardless of the abovementioned qualities, its efficacy in removing paracetamol from water was found to be limited. The percentage of the substance removed from the solution in the experiment was only 12.7%, in comparison with 88.3% for indomethacin and 85.8% for chlorpheniramine maleate. The adsorption process was found to be significantly influenced by pH, in addition to contact time, the quantity of adsorbent, and the concentration of the pharmaceutical compound. In most cases, it was shown that due to an increase in pH, the removal of the active substance from water was reduced. It was estimated that for the majority of examined pharmaceuticals, including paracetamol, the optimum pH was 2.

It is possible to modify the structure of zeolite by means of a cationic surfactant [68]. Such a change allows the surface to become hydrophobic rather than hydrophilic, thereby enabling enhanced adsorption of organic compounds (e.g., paracetamol) and anions. The hexadecyltrimethylammonium ion (HDTMA) was identified as a cationic surfactant comprising a hydrophilic portion, which consists of quaternary ammonium compounds and a hydrophobic portion consisting of a 16-carbon alkyl chain. The adsorption of organic compounds by the zeolite modified with the surfactant was significantly more effective than in the unmodified zeolite.

Clay materials were found to possess a layered structure consisting of a combination of silicate tetrahedra and aluminum hydroxide octahedra [69]. The surface of those materials can be modified in a variety of ways, including acid activation, heat treatment, or chemical modification with a surfactant [70].

In the domain of water treatment, clay emerges as the most economical of the applied adsorbent materials. It is regarded as a safe and cost-effective sorbent for numerous preparations, including paracetamol [71]. In order to enhance its adsorption capacity, it can be modified with distilled water or acid, for example, hydrochloric acid. A thorough analysis of the minerals that underwent that process revealed that they exhibit an enhanced capacity to remove paracetamol. The percentage of positive results obtained was consistently above 80%, even in cases where the active substance was present at low concentrations [70]. Consequently, they can be considered as a potential substitute for costly nanomaterials commonly used in water purification processes. It was shown that acid-modified clay was characterized by higher porosity and a larger specific surface area, thus resulting in much better adsorption properties. However, for optimal efficiency and rapid removal, it is advisable to use both forms concurrently. The materials exhibited optimal adsorption capacity at pH = 6, in a range of 20.4–30.5 milligrams per gram. It is evident that the use of clay, a material that is easily affordable, renders this solution a cost-effective alternative.

Another method for modifying clay involves the incorporation of hexadecyltrimethylammonium bromide (HDTMA) [72]. This improvement was shown to result in a significant increase in adsorption capacity, which was attributed to the formation of novel hydrophobic interactions. The maximum adsorption capacity was found to be 112.63 mg/g. For comparison, the adsorption capacity in unmodified clay was found to be twice as low (62.11 mg/g). A significant advantage of this modified clay was its virtually unchanged adsorption capacity in environments with different pH values, which was due to the neutral nature of the substrate. This property of maintaining a neutral pH is an important aspect in terms of environmental safety.

Saponites are a group of clay minerals belonging to the smectite subgroup. They are characterized by a packet structure consisting of two layers of tetrahedral SiO_4_ and a layer of octahedral MgO_6_(OH)_2_ between them. Kinetic experiments showed that paracetamol exhibits suboptimal adsorption properties on smectite clay [73]. The low affinity of paracetamol for smectite can be attributed to the fact that this compound practically does not enter the interlayer space of smectite, and hence the adsorption is limited only to the outer surface, with interaction mainly through van der Waals forces and the aromatic ring. Materials of this type are characterized by their ion exchange capacity, which renders them suitable for the adsorption of codeine, diazepam and oxazepam. However, in the case of paracetamol, the absence of interactions with cations results in equilibrium adsorption levels of only 20%.

### 3.3. Other Nanomaterials

It is well known that nanomaterials can function as adsorbents for paracetamol, with carbon nanotubes (MWCNT) and graphene (G) being notable examples [74]. The adsorption capacities of the samples were found to be 91.4 mg/g and 18.9 mg/g, respectively. The underlying reason for this discrepancy can be attributed to the increased specific surface area of carbon nanotubes. In addition to the adsorption process, further degradation and desorption of paracetamol were analyzed using ultrasound and gamma radiation (γ) and applying the advanced oxidation processes (AOP). The studies showed that the integration of those phenomena can enhance water treatment processes.

Another effective nanocomposite for removing paracetamol is a combination of titanium oxide (IV) (TiO_2_—titanium white) and activated carbon [75]. This modification resulted in an increase in the surface area and the volume of TiO_2_ pores from 5% to 70%. As previously mentioned, the AOP phenomenon, which includes photocatalysis, was used to degrade pollutants, whereas TiO_2_, due to its high activity, stability, low toxicity and low cost, can be an excellent photocatalyst. However, the material also has serious drawbacks, such as a relatively small specific surface area, which hinders the adsorption of pollutants on its surface, and the fact that it can only be activated by UV radiation. The first drawback could be overcome by combining the material with carbon. The study evaluated the systems with varying proportions, of which the system with an active carbon concentration of 30% (TiO_2_/AC-30) was most effective. This nanocomposite exhibited an approximate photodegradation efficiency of 91% for paracetamol. Furthermore, the research showed that the compound retained its activity even after five cycles, suggesting its potential for repeated use. The study also showed that photocatalytic nanomaterials can be used as compounds for the photodegradation of organic pollutants such as paracetamol.

Mesoporous silica nanoparticles (MSN) are distinguished by their structural characteristics, including the modified morphology, an enlarged surface area, a stable mesostructure, a uniform pore size, a controlled volume, and an adjustable diameter [76]. Furthermore, to enhance their adsorption capacity, the surface is modified with functional groups. The study introduced a pH-sensitive brush polymer, PTBAEMA (poly (2-tert-butylaminoethyl) methacrylate). The polymer undergoes swelling in an acidic environment as a consequence of protonation of the tertiary amine groups located within the polymer chain. Conversely, deprotonation of those groups in an alkaline environment results in polymer collapse. The maximum adsorption efficiency of paracetamol (approximately 92%) for the MSN-PTBAEMA sorbent was obtained within the pH range of 5–7. This phenomenon can be attributed to the π-π interaction between paracetamol and the hydrophobic sites on the surface of MSN-PTBAEMA. An increase in pH to 9 resulted in a decrease in efficiency to approximately 40%, which was due to the collapse of the brush structure and thus a reduction in its surface area. The study showed the efficacy and significance of using this material to remove paracetamol from the aquatic environment.

A nanocomposite that can also be used as an adsorbent material is a copolymer consisting of β-cyclodextrin (β-CD) molecules and nitrogen (N)-doped carbon nanotubes (CNTs) [77]. Cyclodextrins are naturally occurring cyclic oligosaccharides based on glucose. The complexes were observed to form with hydrophobic compounds that are soluble in water, suggesting a possibility of guest-host inclusion processes within those structures. This phenomenon prompted an increased interest among researchers to use those complexes as recyclable paracetamol adsorbents. However, certain modifications are required to achieve optimal functionality. A significant modification involves the conversion from water-soluble compounds to insoluble compounds. In order to achieve this, the copolymerization process was applied with carbon nanotubes (CNTs). This modification also contributed to enhancing the recyclability of the product. Another modification involves increasing the covalent chemistry of CNTs by introducing nitrogen. This process facilitates the anchoring of groups and molecules that are conducive to subsequent functionalization. Still another advancement was the immobilization of iron nanoparticles in CNTs which, due to their reducing and sorption properties, enable the removal of metallic contaminants by converting them into less soluble forms by changing their oxidation state and/or adsorption. The immobilization of those NPs by anchoring them in nitrogen-doped carbon nanotubes prevented them from being washed out into the solution during water treatment. The resulting nanocomposites were found to be both effective and inexpensive adsorbents. The maximum sorption capacity for paracetamol was determined to be 41 mg/g (on N-CNTs-β-CD) and 75.2 mg/g (on Fe/N-CNTs-β-CD). Furthermore, it was observed that the changes in pH of the environment had virtually no effect on the adsorption capacity.

It is evident that both magnetic and zeolite nanomaterials can be used to purify water from paracetamol [78]. The highest efficiency was obtained at pH = 6, following 8 h of contact with the drug. Magnetic nanomaterials proved to be a little more effective, with a removal efficiency of nearly 85% and an adsorption capacity of 68.8 mg/g. In the case of zeolite materials, the efficiency was equal to 75.4% and the adsorption capacity was 49.5 mg/g.

### 3.4. Biocomposites and Materials of Natural Origin

There is an increasing tendency to utilize natural materials in various areas of life, and a similar trend can also be observed in the field of adsorbents. Research findings from the past two decades demonstrate that chitosan-based materials (i.e., chitosan-graphite, SiO_2_–Fe_3_O_4_ or NiFe_2_O_4_–COF–chitosan terephthalaldehyde (NCCT))—initially evaluated for their capacity to remove metals, dyes and pesticides—are being utilized with increasing frequency to address emergent pollutants, such as pharmaceuticals. Machine learning methods have been employed to enhance the efficacy of water treatment processes involving chitosan. Nevertheless, advancement in this domain continues to trail that observed in the field of commercial adsorbents, specifically activated carbon and biochar. The large-scale implementation of technologies based on the use of chitosan necessitates validation in complex real-world conditions, improvement of cost-effectiveness and regeneration efficiency, and reduction in the environmental impact of the extraction and processing of chitosan. It is recommended that the focus be directed towards pilot and field trials, in addition to the improvement of chitosan-based materials for niche applications and the development of “smart materials” that respond dynamically to environmental stimuli. This approach will facilitate the implementation of chitosan-based solutions, ranging from laboratory-scale demonstrations to impactful, scalable innovations in global water treatment [79,80].

Peat is another naturally occurring material that contains lignin and cellulose. These significant components possess the potential to function as a sustainable source of biomass for the production of activated carbon/biochar. The research team of Shukla et al. utilized an aqueous medium on magnetically modified sulphurised peat-based activated carbon (MEPBAC) for the adsorption of caffeine and sulfamethoxazole [81]. As posited by M. Mohammadzadeh and T. Leiviskä, magnetite-pine bark and iron-modified peat have been demonstrated to function as effective, cost-effective, and environmentally friendly biosorbents for the removal of pharmaceutical contaminants with antibacterial properties [82]. Peat, as an adsorbent material, has been extensively studied by various scientists [83]. In the quest for the most effective adsorbent, tannin-rich raw materials were also employed. The bark extract of *Acacia mearnsii* de Wild, *Schinopsis balansae*, *Cupressus sempervivens* and *Pinus pinaster* was utilized in the study. The studies demonstrated the high effectiveness of all of the aforementioned treatments in removing the pharmaceutical substance utilized in the treatment of acute, uncomplicated urinary tract infections [84].

A composite material consists of at least two components that are bonded together at the microscopic level and, concomitantly, at the macroscopic level, thereby exhibiting the properties of a homogeneous material. As the name suggests, biocomposites are products in which one of the components of a heterogeneous mixture is a substance derived from natural sources [85]. Multiple studies were performed on the adsorption properties of biocomposites.

Akköz et al. analyzed the optimal conditions for the adsorption process of paracetamol on Cannabis biomass [86]. The adsorbent under scrutiny was a sulphate biocomposite which had been produced by modifying hemp fibres with H_2_SO_4_ acid. The aim of the analysis was to ascertain the adsorption strength of the biosorbent in the process of purifying an aqueous solution of malachite green. The applied analytical techniques included FT-IR, SEM, EDX, BET, XRD, Boehm titration, and the calculation of the zero charge point and the pH value at which the surface charge of the adsorbent was equal to zero. The study showed that the observed process was spontaneous and endothermic, with very little influence of the changes in ionic strength. The examined material was subjected to a number of adsorption and resorption processes. It was observed that following ten revitalization treatments, there was a significant decrease in purification efficiency of the biocomposite.

The results of the above analyses show a satisfactory adsorption effect of the hemp fibre biocomposite, and also a possibility of repeated use of a single adsorbent [86].

Escapa et al. studied microalgae of the genus *Synechocystis* sp. that can also be used as an adsorbent [87]. A comparison was made between the paracetamol adsorption properties of two substances and those of commercially available activated carbon. This comparison was made according to both the Langmuir and Freundlich isotherm models. In the case of activated carbon, the Langmuir model proved to be much better. However, when it came to describing the biosorption by microalgae, both isotherms yielded satisfactory parameters. For both adsorbents examined, the adsorption properties exhibited an increase with an extended contact time with paracetamol, attaining stability after 240 min. However, microalgae exhibited a substantially lower purification efficiency for the acetophenone-containing aqueous solution, with a mere 10% of the initial mass of the pharmaceutical compound being adsorbed, yielding an adsorption capacity of 53 mg/g. In contrast, carbon exhibited twice as big (20%) a capacity to adsorb the drug, achieving the adsorption capacity of 278 mg/g.

*Microalgae synechocystis* belong to *Merismopediaceae* [88]. Their ubiquity in both saltwater and freshwater environments renders them a viable option for the treatment of pharmaceutical wastewater without causing a significant disruption to the ecosystem. However, it should be noted that there is a risk of disrupting the balance between organisms if too much microalgae is added to the collection. It would be necessary to achieve a quality of treated wastewater comparable to that obtained with activated carbon, as five times more adsorbent would have to be used [87].

The study of Sajid et al. also focused on activated carbon produced from hemp fibres (*Cannabis sativum*), a fast-growing plant that covers roadsides and agricultural wasteland [64]. The obtained adsorbent was then subjected to the chemical activation process using phosphoric acid. The structure that was found to be conducive to the adsorption process (i.e., the structure which had the appropriate surface porosity) was confirmed by means of SEM (scanning electron microscopy). Furthermore, the pH range of 5–7 was identified as the optimal environment for the binding of paracetamol molecules. The obtained data were described using the Langmuir isotherm, which indicates a uniform distribution of sorbent binding centres within the carbon layer. The most favourable kinetic model describing the process was found to be pseudo-second-order kinetics (R^2^ = 0.989). This suggests that the adsorption by chemical bonds is the predominant process in this instance. The maximum adsorption capacity for the activated carbon obtained was 16.18 mg/g [64].

The maximum adsorption capacity of carbon obtained in that study did not show optimal properties for purifying water from paracetamol in comparison with the aforementioned adsorbents. Nevertheless, the low cost of acquisition and the straightforward methods of producing carbon from hemp fibres encourage further research to develop a more efficient biocomposite.

An intriguing approach adopted by scientists involved the investigation of the adsorption potential of plant materials that are typically regarded as waste, including grape stems, yohimbine bark, and cork oak [89]. Surface imaging techniques were used to ascertain the pore structure. The level of paracetamol adsorbed by a given adsorbent was measured by IR and UV-Vis spectroscopy. It is evident from the performed studies that three main factors were identified as influential in determining the binding strength of drug molecules within the pores. They are as follows.

- Interactions are defined as bonds formed between two aromatic rings. These rings are present in both acetophenone and the structure of the plant.
- The formation of hydrogen bonds is a consequence of the presence of amino, hydroxyl and carbonyl groups in the structures of the drug and the adsorbent.
- The hydrophobic effect is the result of the lipophilicity of the interior of the pores of the tested materials and the non-polarity of the benzene ring of the paracetamol molecule [89].

The present study shows the efficacy of natural composites in the treatment of pharmaceuticals in wastewater, while concurrently providing a higher level of recycling for plant waste. However, there is still no evidence to suggest that they are a more effective adsorbent than commercial activated carbon.

## 4. Current Scenarios and Perspectives

Contamination with active pharmaceutical residues is becoming a global problem. The increasing demand for pharmaceuticals has been shown to result in a corresponding increase in both industrial and household waste. Adsorption, a cost-effective and efficient method of wastewater treatment, presents opportunities for industrial application. Research is currently underway to enhance the efficiency, selectivity, and potential for complete recovery of the adsorbent, while concomitantly seeking to minimize deleterious effects on the environment. Nevertheless, even high removal efficiencies, as described in numerous studies, do not fully guarantee stable retention of paracetamol without desorption, particularly in the presence of unstable conditions (e.g., pH, the presence of competing molecules). This suggests that the quality of treated water may be compromised if the behaviour of the adsorbent during regeneration or prolonged exposure is not thoroughly understood. This assertion is corroborated by a comprehensive review of the literature, which encompasses both the efficacy of adsorption processes and the challenges associated with the regeneration of adsorbents in the context of pharmaceutical contaminant removal.

The present focus of research worldwide is on the removal of hazardous pollutants from water and wastewater. Despite the development of a wide range of wastewater treatment methods with the objectives of sustainability, efficiency, feasibility, low cost, and biodegradability, challenges persist in the large-scale production of locally sourced, selective, environmentally friendly, and reusable materials. The overall cost of an adsorbent is influenced by a number of factors, including the necessity for modification, the service life of the product, activation processes, the availability of raw materials, and the potential for reuse.

The focus of contemporary research on paracetamol adsorption is predominantly oriented towards the development of innovative adsorbent composites and biopolymer-based materials. An illustration of this phenomenon can be observed in the utilization of ZnO nanoparticle-immobilized chitosan-inulin composites [90]. The material demonstrated high reusability, maintaining effective performance for up to 13 cycles, affirming its potential for repeated applications in removing paracetamol from aqueous systems.

A relatively recent solution involves the use of biological materials to remove pollutants from wastewater, i.e., biosorbents. The level of interest among scientists in this material has increased considerably. In order to enhance their capacity to eliminate pharmaceutical compounds from water systems, a number of biosorbents have been modified. The focus on biosorbents as materials for use in water treatment can be attributed to several factors. These include their natural origin, biodegradability, ease of modification and origin from renewable resources. Furthermore, the utilization of waste products as raw materials for the fabrication of biosorbents has been documented [91]. Algae, specifically *Scenedesmus obliquus*, have been successfully utilized as an adsorbent for the removal of selected pharmaceutical compounds from water, including paracetamol, ibuprofen, tramadol and ciprofloxacin [92]. Another biosorbent that gained considerable popularity at the end of the 20th century, mainly due to various environmental concerns, is sisal waste. This is chemically activated to produce activated carbon, which has enormous potential for removing ibuprofen and paracetamol [93]. The adsorption process of pharmaceuticals was also studied using acid-treated cuttlefish bone powder [94].

Another emerging trend pertains to the utilization of sustainable, cost-effective precursors, such as agricultural waste, for the production of activated carbon. An exemplar of this approach is biochar [95]. In the last decade, research into biochar application has emerged as a significant topic in the field of water treatment studies, thus establishing biochar adsorption as one of the primary wastewater treatment strategies. Biochar is a porous solid consisting primarily of amorphous carbon, which is obtained from biomass materials through the process of pyrolysis. In recent decades, biochar has been the subject of extensive research, with a view to ascertaining its potential applications as a durable soil enhancer and an environmental remediator. It is recommended that future research efforts concentrate on the optimization of various parameters within the production process of biochar, with a view to enhancing its adsorption capacity.

In the context of this research, it should be emphasized that contemporary studies include detailed mechanistic and kinetic analysis, often with advanced modelling. Mechanistic investigations have been identified as a key focus in the recent literature. For instance, in the study of ZnO/chitosan-inulin nanocomposites, adsorption is described using statistical physics models, Langmuir isotherms, fractal kinetics, and intra-particle diffusion analysis, thereby facilitating a more profound comprehension of adsorption mechanisms, energetics, and surface heterogeneity. Furthermore, a significant body of research has been dedicated to the examination of pseudo-second-order kinetics and isotherm modelling (Langmuir/Freundlich) for the purpose of quantifying the adsorption behaviour of paracetamol on a variety of adsorbents [96].

The final significant task to be addressed by contemporary methodologies, signifying prospective advancements, pertains to the emphasis on adsorbent regeneration and reuse for real-world viability. Additionally, there is a need to consider natural materials, such as zeolites and clays, as potential alternatives to engineered adsorbents. The objective of these approaches is to enhance the environmental sustainability of purification methodologies. In the case of paracetamol, the regeneration of adsorbents should be particularly developed, as specific paracetamol studies on regeneration are not as abundant. This trend has been extensively documented in reviews of adsorbent desorption and reusability patterns, particularly in the context of biopolymeric and hydrogel adsorbents employed in pharmaceutical adsorption. Consequently, significant challenges are anticipated for the future implementation of paracetamol in practical applications.

A contemporary approach that has seen a marked progression in technological development is the utilization of computerized methodologies for the modelling of adsorption processes. This encompasses the employment of machine learning algorithms and neural networks, which have been shown to facilitate increasingly precise and comprehensive applications. The novel strategy for designing porous adsorbents using this approach has great potential to facilitate the production of novel carbon adsorbents that are optimized for the purification of aqueous solutions from non-electrolyte (like paracetamol) contaminants [57,97,98,99,100].

## 5. Conclusions

In recent years, ecotoxicology and the removal of pharmaceutical pollutants have been the subject of numerous scientific and review articles. Collectively, these works have contributed to the advancement of knowledge in the field of pharmaceutical wastewater treatment systems. It is proposed that research should focus on the ecotoxicological impact of pharmaceutical pollutants in mixtures, the realization of ecotoxicological research conditions, and the consideration of interactions between pharmaceuticals. The mixture of different pollutants also poses a challenge in the design of adsorbents due to the distinct functional groups in the structure. The adsorption efficiency for a given target compound may be subject to variation depending on the nature of the adsorbent. The synthesis of environmentally friendly materials containing different functional groups that can adsorb multiple pollutants may be a topic of interest for future research. The extant research results primarily concern purification processes and their effectiveness. The toxicity assessment of wastewater that has already undergone treatment is a subject that is rarely discussed. Notwithstanding the treatment of water, it may still be toxic to the environment, for example, due to secondary contamination by the adsorbent or by-products of the treatment process. In order to ensure the safety of the patient, it is recommended that the treatment process be complemented by toxicity tests.

The review confirms that adsorption is an effective method of removing paracetamol from wastewater. Of the materials analyzed, activated carbon, especially when chemically modified or derived from lignocellulosic waste, exhibits the highest adsorption efficiency, often achieving removal rates above 90%. Although nanomaterials such as carbon nanotubes, graphene and TiO_2_-based composites were found to increase the surface area and can improve the adsorption and photodegradation, they are not widely used due to high cost and regeneration challenges.

Zeolites and clays are a more economical alternative, but their adsorption capacities are generally lower unless their structures are modified, for example, by surfactant treatment or acid activation. Biocomposites and natural materials, including hemp-derived products, microalgae and plant waste, were found to be promising and worth considering as environmentally friendly adsorbents. However, further research is required to match the effectiveness of commercial materials.

The efficiency of adsorption depends on various factors, including surface chemistry, pore structure, pH, and the presence of functional groups that facilitate interactions such as π–π stacking, hydrogen bonding and hydrophobic effects. Regeneration and reuse of adsorbents remain critical areas in need of improvement. Of the regeneration methods tested, vacuum annealing appears to offer the most promising results for restoring the adsorption capacity of carbon-based materials.

In conclusion, adsorption emerges as a scalable and environmentally sound approach to paracetamol removal. Further studies should therefore be performed and focus on optimizing production processes, reducing material costs and incorporating adsorption into integrated, multi-stage water purification systems, with the aim of enhancing the overall effectiveness and sustainability of pharmaceutical wastewater treatment.

## Figures and Tables

**Table 1:** List of advantages and disadvantages of activated carbon, zeolites, clays, nanomaterials, biocomposites and bio-based adsorbents based on [34,35,36,37,38,39,40,41].

Adsorbent Class	Advantages	Disadvantages
Activated Carbon	Well-developed porosity and adsorption capacity (especially for organics) Established commercial technology with many available grades	Low selectivity for specific contaminants Difficult regeneration without loss of capacity
Zeolites	High selectivity for certain species (e.g., NH_4_^+^, heavy metals) Microporous structure with ion-exchange capacity Thermal and chemical stability	Lower surface area than activated carbons Efficiency depends on zeolite type and pretreatment (some require modification to improve adsorption)
Clays	Low cost, abundant and available materials	Lower surface area than activated carbons Often require modification to improve adsorption
Nanomaterials	High surface area and capacity Rapid kinetics Functionalization leads to more selective adsorbates	Problems with recovery and separation after use
Biocomposites	Often are biodegradable and leave lower environmental footprint	Regeneration is difficult and may cause a decrease in adsorption capacity Problems with stability (structural and/or mechanical)
Bio-based materials	Obtained from renewable sources Low cost	May require activation or modification to improve adsorption efficiency Lower surface area and mechanical strength compared to modified adsorbents

**Table 2:** Comparison of adsorption capacity, effect of pH on adsorption efficiency and relative cost of selected adsorbents [34,38,42,43,44,45,46,47,48,49].

Adsorbent Class	Adsorption Capacity	Effect of pH on Adsorption Efficiency	Relative Cost
Activated Carbon	It can vary widely—often high values (e.g., up to several hundred mg/g for organic contaminants. Activated carbon has a BET surface area > 1400 m^2^/g, which gives it high efficiency).	In the case of organic compounds, adsorption often increases with increasing pH, but depends on the specific adsorbate.	Moderate to high cost—production is relatively energy-intensive (activation, regeneration requirements, and raw material quality).
Zeolites	Values depend on contamination—e.g., in the case of dyes/metals in advanced composite materials, values of 53–409 mg/g have been reported.	pH has a significant effect on ion exchange and surface interactions. The optimal pH depends on the type of contamination and zeolite (for many ions, it is usually close to neutral).	Low to moderate costs—Depending on origin. Natural zeolites are inexpensive, but modified zeolite composites are more expensive due to the need for processing.
Clays	Natural (unmodified) clays often have a lower capacity (e.g., ~100 mg/g for dyes, and even less for metals).	pH can affect surface charge and adsorption: adsorption may increase above the zeta point, but patterns vary depending on the type of clay.	Low cost—widely available natural materials.
Nanotubes	Wide range—from several dozen to several hundred mg/g depending on contaminants. Composite nanotubes may exhibit even higher performance.	The pH value affects the surface charge of nanotubes. For many ions/organic substances, better performance is often achieved near neutral pH.	High cost—synthesis and purification are expensive. Additionally, functionalization increases costs but improves performance.
Nanodots	Potential yields up to ~2000 mg/g, although specific data varies significantly.	The pH value can have a strong influence on the functional groups on the surface of the nanoparticles, and thus on the binding efficiency. Composites are often tailored to specific pH ranges.	Moderate to high cost, depending on the synthesis method.
Biocomposites	Up to hundreds of mg/g depending on the product; some cellulose-biopolymer composites achieve >200 mg/g.	pH affects the ionization of functional groups and the surface charge of the composite.	Low to moderate costs—depending on the origin of the raw materials and the costs of composite synthesis.
Natural Products	Low to moderate yield—e.g., fruit peels, rice husk biochar often <20–50 mg/g	Often pH-dependent due to natural functional groups.	Very low cost—large amount of biomass waste available locally

**Table 3:** Comparison of raw materials used for the production of activated carbon for paracetamol adsorption.

Raw Material	Method of Activation	Adsorption Capacity for Paracetamol [mg/g]	Adsorption Model	Reference
Walnut nutshell	KOH at 750 °C	~435	Langmuir, pseudosecond order	[60]
Pistachio shell	KOH at 750 °C	~330	Langmuir, pseudosecond order	[60]
Peanut shell	KOH at 750 °C	~350	Langmuir, pseudosecond order	[60]
Biomass waste	KOH activation	~355	Langmuir, pseudosecond order	[61]
Sludge-derived activated carbon	Acid-treated beverage sludge pyrolyzed	~145	SIPS model	[62]
Palm leaves waste	KOH activation	~90	Langmuir, pseudosecond order	[63]
Cannabis sativa hemp	H_3_PO_4_ activation	~15	Langmuir, pseudosecond order	[64]
Dende and Babassu coconut mesocarp	chemical activation	~70	Langmuir	[65]

## Data Availability

No new data were created or analyzed in this study.
